# Antagonizing RARγ Drives Necroptosis of Cancer Stem Cells

**DOI:** 10.3390/ijms23094814

**Published:** 2022-04-27

**Authors:** Geoffrey Brown

**Affiliations:** School of Biomedical Sciences, Institute of Clinical Sciences, College of Medical and Dental Sciences, University of Birmingham, Edgbaston, Birmingham B15 2TT, UK; g.brown@bham.ac.uk; Tel.: +44-(0)121-414-4082

**Keywords:** cancer stem cells, oncogenes, retinoic acid receptors, prostate cancer, necroptosis

## Abstract

There is a need for agents that eliminate cancer stem cells, which sustain cancer and are also largely responsible for disease relapse and metastasis. Conventional chemotherapeutics and radiotherapy are often highly effective against the bulk of cancer cells, which are proliferating, but spare cancer stem cells. Therapeutics that target cancer stem cells may also provide a *bona fide* cure for cancer. There are two rationales for targeting the retinoic acid receptor (RAR)γ. First, RARγ is expressed selectively within primitive cells. Second, RARγ is a putative oncogene for a number of human cancers, including cases of acute myeloid leukemia, cholangiocarcinoma, and colorectal, renal and hepatocellular carcinomas. Prostate cancer cells depend on active RARγ for their survival. Antagonizing all RARs caused necroptosis of prostate and breast cancer stem cell-like cells, and the cancer stem cells that gave rise to neurospheres from pediatric patients’ primitive neuroectodermal tumors and an astrocytoma. As tested for prostate cancer, antagonizing RARγ was sufficient to drive necroptosis. Achieving cancer-selectively is a longstanding paradigm for developing new treatments. The normal prostate epithelium was less sensitive to the RARγ antagonist and pan-RAR antagonist than prostate cancer cells, and fibroblasts and blood mononuclear cells were insensitive. The RARγ antagonist and pan-RAR antagonist are promising new cancer therapeutics.

## 1. Introduction

An often-asked question is will there ever be a cure for cancer? Cancer is a group of diseases and moreover, and for every cancer, variant clones evolve in a Darwinian manner as the disease progresses, including clones that are resistant to chemotherapeutics [1]. Potentially, chemotherapy provides a selective pressure that leads to the expansion of drug-resistant variants [2]. These matters lie at the heart of the failure to cure many cancers. The challenge is to either sustain control of disease for the rest of a patient’s life and/or to increase the chances of achieving a cure at disease presentation and the onset of treatment. Regarding both of these options, the cells that sustain a cancer are cancer stem cells (CSCs) which produce the hierarchy of cells for cancer. Their frequency within cancer varies from exceedingly rare, as for human acute myeloblastic leukemia (AML) [3], to up to 25%, as for human melanoma [4]. For many solid cancers, the nature and frequency of CSCs are still uncertain. CSCs are also largely responsible for disease relapse and metastasis, and the treatment of metastasized cancers has not advanced significantly and often they are beyond successful treatment. To increase the chances of providing a *bona fide* cure for cancer there is the need to develop a strategy to control and/or eliminate CSCs.

The failure of conventional treatments to eliminate CSCs is well illustrated by studies of chronic myeloid leukemia (CML). This leukemia arises from the transformation of a hematopoietic stem cell (HSC) [5], and it has been known since 1999 that CML leukemia stem cells (LSCs) are insensitive to high doses of chemotherapeutic agents that target the cell cycle [6]. A subpopulation of primitive CML leukemia cells that were highly quiescent LSCs was isolated from patients with chronic-phase CML and were insensitive to high doses of chemotherapeutic agents that are efficacious against the dividing leukemia cells. Additionally, tyrosine kinase inhibitors, such as imatinib, are used to treat CML and many different types of cancer. In vitro, CML LSCs were observed to be insensitive to imatinib, and, are presumed to be insensitive in vivo [7,8]. The challenge to curing CML is one of eliminating the LSCs that are spared by current treatments. CML LSCs can also cause disease relapse even after allogeneic transplantation [9].

There are substantial efforts to develop the means to eradicate CSCs [10,11]. The approaches include chimeric antigen receptor T cells (CAR T cells), antibodies, and small molecules that target CSCs. For example, the premise in CAR T cell targeting is that the molecules recognized are over-expressed on cancer cells and at a low level on normal cells. As yet, successes from the use of CAR T cells are rare, because surface antigen expression by cancer cells is highly heterogeneous and the need to identify a proper target for each patient, including even for patients with the same type of cancer [12]. Heterogeneity of antigen expression by cancer cells and whether the antigen is expressed by normal cells are also concerns regarding antibody targeting of CSCs. A general approach to developing anticancer drugs is to interfere with the intracellular events that regulate the survival of cancer cells. Moreover, it is important to eliminate CSCs without too much damage to normal stem cells. This review focuses on targeting RARγ to eliminate CSCs by the use of an antagonist to switch-off RARγ.

## 2. Why Target RARγ to Eliminate CSCs?

The three main isotypes of RAR are RARα, RARβ, and RARγ. They form dimers with members of the retinoid X receptor subfamily which bind to response elements to act as ligand-regulated transcription factors. The ligand for RARs is all-*trans* retinoic acid (ATRA), which is the major bioactive metabolite of retinol or vitamin A. Disruption of ATRA signaling is thought to play a role in the etiology of many cancers. The list includes leukemias, breast cancer, glioblastoma, head and neck cancer, liver cancer, lung cancer, ovarian cancer, neuroblastoma, pancreatic cancer, prostate cancer (PCa), renal cell cancer, and skin cancer [13]. ATRA is a potent pro-differentiation, anti-proliferation, pro-apoptosis agent and its therapeutic use has provided a cure for acute promyelocytic leukemia (APL) [14]. APL accounts for 5–15% of cases of AML and is classified as AML M3 under the French-American-British (FAB) system. The hallmark signature of APL is the t(15;17)(q24;q21) translocation that fuses the *PML* and *RARA* genes leading to the expression of the oncogenic PML-RARα protein. There is evidence to support the view that APL arises in an HSC from the presence of the PML-RARα protein in patients’ LSCs [15]. ATRA targeting of both PML-RARα and wild type RARα results in the dissociation of transcriptional corepressors, proteolytic degradation of PML-RARα and wild type RARα, and differentiation and apoptosis of APL cells. The therapeutic use of a combination of ATRA and arsenic trioxide has led to long-lasting disease remission, and in this regard, greater demethylation of genes may be important [16]. Unfortunately, the success of ATRA in providing differentiation therapy for APL has not translated to other cancers.

### 2.1. The Role of RARγ within Stem Cells

There are three rationales for targeting RARγ to eliminate CSCs. RARγ is selectively expressed within stem cells and their immediate offspring, it plays a crucial role in the survival of these cells, and is an oncogene for a number of cancers. The selective expression of RARγ and its role are well described for HSC development and RARγ and RARα have discrete physiological roles. Active RARγ promotes HSC survival and self-renewal, whereas active RARα promotes differentiation, and this balance is critical to the proper conduct of hematopoiesis. RARγ expression is restricted to primitive hematopoietic cells and ATRA-activated RARγ supports HSC self-renewal as knockout mice had a reduced number of HSCs [17]. It is well established that ATRA-activated RARα drives the terminal maturation of committed granulocyte/monocyte progenitors [18].

Studies of the early development of zebra fish embryos have provided further support to the fact that RARγ plays a crucial role in stem cells. Zebra fish embryos were treated in vitro with a RARγ-selective agonist. RARγ activation blocked stem cell development, preventing fin, bone and neural ganglia development. Stem cell numbers were unaffected because wash-out or the use of a RARγ antagonist to reverse the action of the RARγ agonist restored fin formation. In this case and in the absence of ATRA, RARγ functions to maintain stem cells [19]. Studies of RARγ knockout embryonic stem (ES) cells have also revealed the importance of RARγ to stem cells. For ES cells, ATRA-regulated transcripts are dependent on a functional RARγ and, therefore, RARγ is essential for transcriptional activation in ES cells. The studies also revealed that RARγ is essential for chromatin remodeling and DNA epigenetic marks [20]. It is also important to bear in mind that RAR/retinoid X receptor dimers bind to genomic regions that are characterized by the binding of pluripotency-associated factors [21].

### 2.2. RARγ Is an Oncogene for a Number of Cancers

The need is to spare normal stem cells whilst eliminating CSCs. In this regard, RARγ is an oncogene for a number of cancers. As mentioned above, APL is characterized by *RARA*-associated gene rearrangements. There is dysregulation of the RARG gene in APL-like leukemia. Fusions have been identified between the *RARG* gene and the genes for PML, CPSF6 (a subunit of the RNA binding protein cleavage factor 1), NPM1 (nucleophosmin), and NuP98 (nucleoporin) [22,23,24]. These patients did not respond to ATRA treatment. For an APL-like patient that lacked a *RARA* rearrangement, a reciprocal fusion involving *RARG* and *HNRPC3* (heterogeneous nuclear ribonucleoprotein C) has been reported. The patient was treated with ATRA and arsenious acid, arsenious acid was withdrawn because a *RARA* rearrangement was lacking, and there was no response to ATRA [25]. The potential impact of RARγ expression on disease is illustrated by a patient with relapsed AML who died from rapid disease progression after ATRA treatment. An increase in the level of nuclear RARγ was observed when primary cells from this patient were treated with ATRA in vitro, which may explain the rapid disease progression in response to ATRA therapy [26].

From quantitative PCR and Western blotting studies, RARγ mRNA and protein are frequently overexpressed in human colorectal cancer (CRC) tissue versus the surrounding non-tumorous colorectal tissue. Similarly, expression of RARγ is increased in the CRC cell lines HT29, HCT116, RKO and SW480 as compared with the HCoEpiC normal colonic epithelial cells. For the HT29, HCT116, and RKO cell lines, knockdown of RARγ enhanced their sensitivity to 5-fluorouracil, oxaliplatin, and vincristine. This was found to be related to a decreased expression of the multi-drug resistance 1 protein. RARγ is, therefore, a potential therapeutic target for chemotherapeutic resistance CRC [27]. Similarly, overexpression of RARγ in the bile duct carcinoma cholangiocarcinoma (CCA) is associated with a poor prognosis and resistance to 5-fluorouracil. Knockdown of RARγ expression in the three human CCA cell lines QBC939, SK-ChA-1, and MZ-ChA-1, by siRARγ, resulted in the suppression of cell proliferation. Colony formation and xenograft tumor growth in nude mice were reduced in the case of QBC939 cells that were stably transfected [28]. RARγ appears, therefore, to be important for CCA tumorigenesis. The majority of primary tissue samples from patients with hepatocellular carcinoma (HCC) overexpress RARγ, as do HCC cell lines. For the HCC cell line HepG2, colony formation and xenograft engraftment were promoted by overexpression of RARγ [29]. From the use of qPCR and bioinformatics analyses, around 50% of tissues from patients with clear cell renal cell carcinoma were observed to overexpress RARγ [30].

### 2.3. PCa Cells Are Dependent on Active RARγ for Their Survival

**Figure 1:**
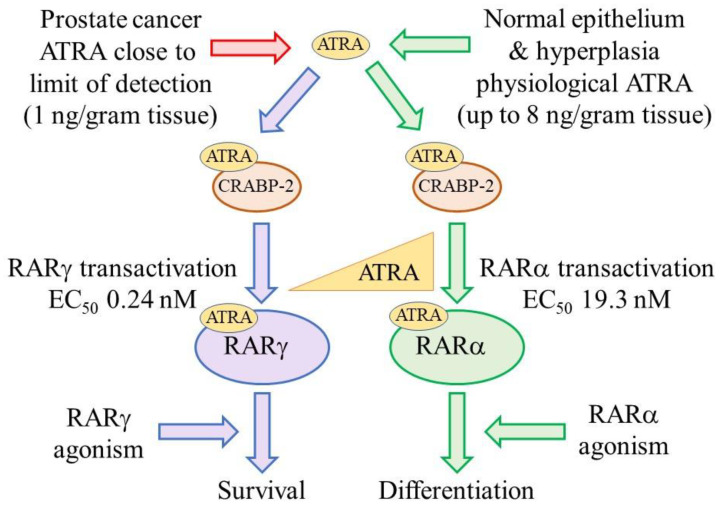
Patients PCa cells survive and grow in an abnormally low level of ATRA. The low level of ATRA within PCa tissue is important because 0.24 nM ATRA transactivates RARγ, whereas RARα transactivation requires a much higher concentration of 19.3 nM ATRA. Patients’ PCa cells are, therefore, reliant on RARγ transactivation for survival and proliferation. From various studies, active RARγ is pro-survival whereas active RARα is pro-differentiation.

Patients’ PCa cells and normal prostate epithelium express RARα and RARγ. The key finding that supports the view that RARγ is an oncogene in PCa is that PCa cells depend on activated RARγ for their survival as follows. Patients’ PCa cells survive and grow in an abnormally low level of ATRA (Figure 1) because the level in patients’ tissue was observed to be very close to the limit of detection, at around 1 ng/gram tissue. The level in the surrounding normal tissue and benign prostate hyperplasia is up to 8 times higher [31]. It has been reported that RARγ has a constitutive closed helix 12 conformation that interferes with corepressor recruitment and that there is a level of target gene activation in the absence of ATRA [32]. Even so, transactivation studies have shown that 0.24 nM ATRA (equivalent to 1 ng/gram tissue) transactivates RARγ, whereas RARα transactivation requires a much higher concentration of 19.3 nM ATRA [33]. The low level of ATRA in patients’ PCa tissue is important because it is likely that just RARγ is transactivated in PCa cells which are, therefore, reliant on active RARγ for survival and proliferation.

There are a number of possibilities regarding the abnormally low level of ATRA within PCa tissue. There is decreased expression of the aldehyde dehydrogenase isoforms that convert retinol to ATRA within malignant prostate cancer tissue [34] and the PCa cell line LNCaP [35]. Dietary vitamin A is transported in the bloodstream as hydrophobic retinol and solely by the retinol-binding protein (RBP). The integral membrane receptor STR6 recognizes RBP-retinol and mediates cellular retinol uptake by triggering the release and internalization of retinol [36]. Induction of the expression of STR6 by retinol and ATRA has been shown to be defective with PC3 cells, compared to normal prostate epithelium. This might reduce the efficiency by which PCa cells can sequester retinol from the environment. Retinol is also metabolized by PCa cells to retinyl esters for storage by lecithin:retinol acyltransferase. The activity of this enzyme is required for the uptake of an appropriate amount of retinol by cells and the level of expression of mRNA was found to be reduced in PC3 cells [37].

## 3. Agonists and Antagonists of RARs

Antagonists of RARs were developed in the late 1990s [38], and the synthetic retinoids developed included antagonists, and agonists, that are highly selective for RAR subtypes. A pharmacological level of ATRA (10^−6^ M) is often used to reveal activity against carcinoma cell lines. Hence, there was interest in whether RAR antagonists are more effective than ATRA against carcinoma cells. As determined by the [^3^H]-ATRA displacement method [39], the equilibrium binding affinities for the new retinoids for their receptor are in the nM range [40], Table 1. They were used at down to nM levels to test their biological activities. Carcinoma cell lines had been adapted to grow long-term in a serum-free medium to avoid any positive or negative effects of the ATRA that is present in serum.

**Figure 2:**
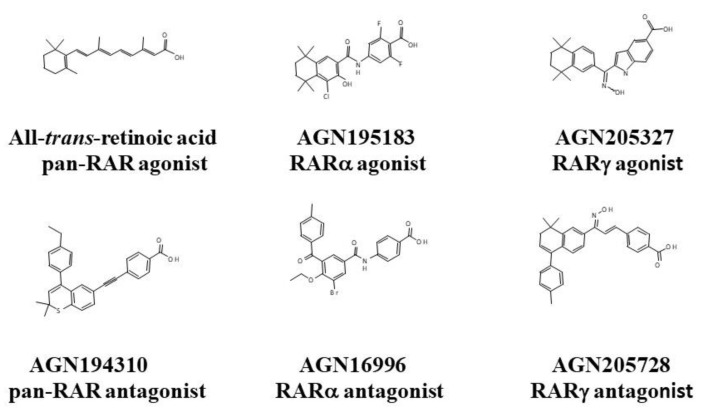
Structures of the RAR agonists and antagonists.

The pan-RAR and RARγ antagonists are of particular interest regarding the killing of CSCs and their structures, alongside that of a RARα antagonist which does not kill CSCs, are shown in Figure 2. Agonists are shown for comparison.

## 4. Antagonizing RARγ Kills CSCs

PCa was a focus of attention in testing the potential therapeutic use of RAR antagonists. It is a complex disease whereby in the first instance there is aberrant differentiation and hyperproliferation of the prostate epithelium. This is followed by malignant transformation, an alteration of the cellular mechanisms to favor an increased survival of malignant cells, and the eventual appearance of genetically diverse malignant clones which are metastatic [41]. At an early stage of the disease, the majority of patients have excessive androgen production which is also often associated with aberrant androgen receptor (AR) signaling [42]. These events are the major drivers of disease progression. Patients with localized disease, a low/intermediate risk of recurrence, and who are diagnosed and treated at an early stage have a 99% overall survival for 10 years [43]. A major pharmacological treatment for prostate cancer is androgen ablation, using androgen synthesis inhibitors and/or AR antagonists [44]. However, and in the majority of cases, an aggressive disease develops that does not respond to androgen ablation, termed castration refractory PCa. These patients often respond poorly to chemotherapy and other pharmacological interventions [45]. There is a need to develop new treatments for PCa.

The human DU-145, LNCaP, and PC-3 cell lines are widely used in PCa research and were derived from metastatic disease [46]. AR variants have been identified in the human PCa cell line LNCaP [47] and the human PCa cell lines DU-145 and PC-3 have an apparently normal AR gene [48,49]. These three cell lines express AR protein, the levels are lower in DU-145 and PC-3 than in LNCaP, and increases in the levels of AR protein were observed in response to dihydrotestosterone [50].

Table 2 shows the potency of pan-RAR and RARγ antagonists against the PCa cell lines and patients’ cells when grown as flask cultures [51,52,53]. The pan-RAR antagonists AGN194310, AGN19309, and AGN193776 were highly effective in inhibiting the growth of the three PCa cell lines (IC_50_ values from 3.5 to 6.8 × 10^−7^ M), AGN194310 was equally effective against patients’ PCa cells (IC_50_ value 4.7 × 10^−7^ M), and normal prostate epithelium was less sensitive to the AGN194310 antagonist than the PCa lines (IC_50_ value 1.0 × 10^−6^ M) [51,52,53]. The pan-RAR antagonist LG100815 is not structurally related to the AGN antagonists. It was a little less effective than the AGN antagonists against the PCa lines, and normal prostate epithelium was less sensitive than the PCa lines. Antagonism of RARγ, by AGN205728, was sufficient to growth arrest the PCa cell lines (IC_50_ values from 3.0 to 6.0 × 10^−7^ M) and primary cells from a PCa patient (IC_50_ value 3.0 × 10^−7^ M) [53]. Normal prostate epithelial cells and the non-malignant RWPE-1 cells were less sensitive to the RARγ antagonist (IC_50_ values of 7.2 × 10^−7^ M and 2.3 × 10^−6^ M, respectively). The RARα antagonist AGN196996 and RARβ antagonist LE135 [54] did not affect the growth of the PCa lines. ATRA and the non-hydrolysable pan-RAR agonist TTNPB [55] inhibited the growth of the three PCa lines at concentrations >10^−8^ M. The proliferation of LNCaP cells was markedly increased by exposure to 10^−10^ to 10^−8^ M ATRA, TTNPB, and the RARγ agonist AGN205327. The RARγ agonist inhibited adipogenic differentiation of PCa cells [53]. From all of the above, RARγ agonism stimulates growth and inhibits the differentiation of PCa cells, and RARγ antagonism drives growth arrest.

The cells that give rise to large colonies when PCa cell line cells are dispersed in a petri dish are CSC-like cells. Table 3 shows the potency of pan-RAR and RARγ antagonists against these cells. The pan-RAR antagonist AGN194310 and the RARγ antagonist AGN205728 were potent inhibitors of colony formation by the DU-145, LNCaP, and PC-3 cell lines [51,52,53]. The IC_50_ values obtained for the pan-RAR antagonist AGN194310 and the RARγ antagonist AGN205728 were between 16 to 34 × 10^−9^ M and 50 to 60 × 10^−9^ M, respectively. The RARα antagonist AGN196996 did not affect colony formation, and the IC_50_ values obtained for ATRA were between 3.2 to 4.2 × 10^−7^ M. The pan-RAR antagonist AGN194310 was equally effective in preventing colony formation by the breast cancer cell lines MCF7 and MDA-MB-231 which were derived from metastatic disease. Other workers have also shown that pharmacological or genetic blockage of RARγ signaling drives growth arrest, differentiation, and cell death of breast cancer cells [56,57].

For LNCaP cells plated in the absence of ATRA, around 70% of the colonies had a large and holoclone/merclone morphology, and these colonies contain stem cells [58]. The remaining colonies had a small and differentiated paraclone morphology. Treatment of LNCaP cells with 10^−10^ M ATRA, to activate RARγ, increased the proportion of stem cell-like colonies to 85%, whereas 10^−6^ M ATRA increased the proportion of differentiation-committed and paraclone colonies to 60% [53]. The low level of ATRA seen within PCa tissue via transactivating RARγ would, therefore, increase the population of clonogenic and stem cell-like cells or block differentiation of these cells.

Treatment of flask-grown cultures of the PCa cell lines with the pan-RAR antagonist AGN194310 and the RARγ antagonist AGN205728 led to growth arrest in G_1_ of the cell cycle followed by cell death. The cell death was mitochondria depolarization-dependent, and involved cellular DNA fragmentation, but was caspase-independent [52,53]. This form of cell death is termed necroptosis and was seen for Jurkat T leukemia cells that were deprived of retinoids. This led to the activation of the poly(ADP-ribose) polymerase PARP-1 which ribosylates a wide variety of proteins, including those involved in transcription and cell cycle, to change their function [59]. Caspase-independent cell death has also been seen for ischemia-reperfusion injury, diabetes, inflammatory-mediated injury, and neurotoxicity [60]. Cell stress activation of PARP-1 has been associated with mitochondrial dysfunction with the release of ATP, NAD^+^ and the caspase-independent nucleases AIF and endonuclease G. They fragment DNA. Inhibition of PARP-1, by 1,5-dihydroisoquinoline (at 1 × 10^−4^ M), blocked the actions of RAR antagonists on the PCa cell lines.

## 5. Antagonizing All RARs Is Effective against Pediatric Brain Tumors

The most common cause of cancer mortality in children are tumors of the peripheral and central nervous system. Post-treatment, a significant proportion of patients have life-long and induced neurological, cognitive and endocrine disturbances [61]. Several of the peripheral and central nervous system tumors share an embryological origin in the neuroectoderm and have been grouped as primitive neuroectodermal tumors (PNETs). They include neuroblastoma, Ewing’s sarcoma, retinoblastoma, medulloblastoma, and supratentorial primitive neuroectodermal tumors (stPNETs) [62]. ATRA has been implicated in the development of the central nervous system [63]. Oral 13-*cis* retinoic acid is a key component of the therapy for neuroblastoma as a consolidation treatment [64], and ATRA has been shown to inhibit the proliferation of human PNET cells [65]. Exploration of the effectiveness of the pan-RAR antagonist AGN194310 was driven by the use of 13-*cis* retinoic acid to treat neuroblastoma requires a high serum level and is severely limited by toxicity.

**Figure 3:**
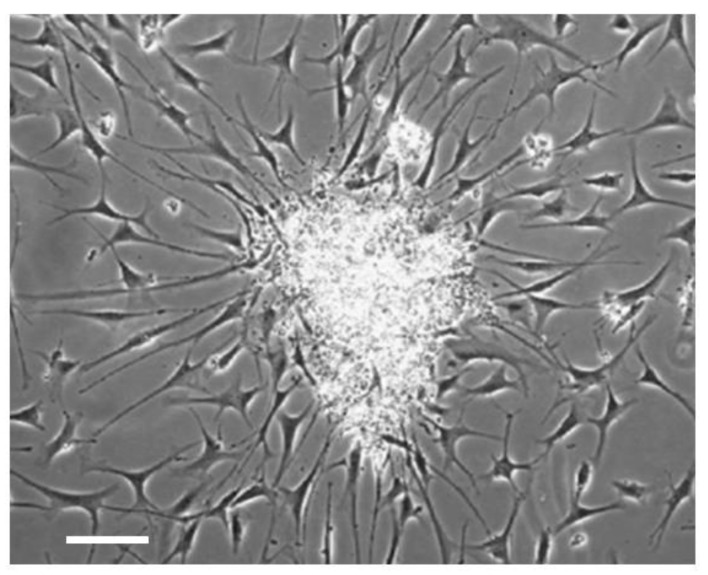
Primary culture of cells from PNET biopsies generates neurospheres with differentiating cells. The cells that give rise to neurospheres are CSCs. Scale bar = 100 μm.

When cells from PNET patient biopsies were cultured in a serum-free neural stem cell medium (Neurocult) supplemented with 20 ng/mL epidermal growth factor they generated neurospheres which produced differentiated cells that migrated (Figure 3). The cells that give rise to neurospheres are CSCs. The activity of the pan-RAR antagonist AGN194310 was examined against two pediatric PNETs and a pediatric astrocytoma. These cells were plated into wells, treated with AGN194310, and cellular ATP levels were measured on day 5. AGN194310 was highly effective against the two pediatric PNETS and the pediatric astrocytoma. Neurospheres and their progeny were completely ablated by 10^−6^ M AGN194310. ATRA and the RARα antagonist AGN195183 were somewhat ineffective [53].

## 6. The Effect of Antagonizing RARs on Normal Cells

Selectivity is all important to killing CSCs and sparing normal tissue cells as much as is possible. As mentioned above, normal prostate epithelium cells were less sensitive to the pan-RAR antagonist AGN194310 and RARγ antagonist AGN205728 than PCa cell lines and patients’ cells. The antagonists did not have an effect on human peripheral blood lymphocytes and primary cultures of human fibroblasts.

An important matter is that we expected the pan-RAR to influence the differentiation of HSCs and/or hematopoietic progenitor cells (HPCs) because active RARα is required for the differentiation of neutrophil/monocyte progenitors [18]. We investigated this by using flask cultures of purified human bone marrow HSCs/HPCs (CD34^+^). Indeed, treatment of these cells with the pan-RAR antagonist AGN194310 and the RARα antagonist AGN196996 prolonged the lifespan of cultures, up to 55 days, and there was a substantial increase in the production of neutrophils and monocytes. This was not related to cell differentiation slowing down, and instead there was an expansion of the number of HSCs and HPCs [33]. From these findings, the use of the pan-RAR antagonist AGN194310 to drive an increase in neutrophil production may be of benefit to patients with neutropenia, including those with chemotherapy-provoked neutropenia.

## 7. Concluding Remarks

PCa cell line CSC-like cells and primary cultures of cells from PCa patient biopsies were ablated by treatment with the RARγ antagonist AGN205728 or the pan-RAR antagonist AGN194310. Normal prostate epithelium cells were less sensitive to the actions of the two antagonists, and human peripheral blood lymphocytes and primary human fibroblast were unaffected. The two compounds were also equally active against breast cancer cell line CSC-like cells and the pan-RAR antagonist AGN194310 was effective against PNET CSCs. The use of a metastatic model of epidermal growth factor receptor-mutant lung cancer has shown that a pan-RAR antagonist dramatically reduces lung cancer metastasis to the brain [66]. Cancer patients receiving intensive chemotherapy often develop neutropenia. The pan-RAR antagonist AGN194310 as used as an adjunct to chemotherapy may provide additional therapeutic benefit because it increases the production of neutrophils by HSCs/HPCs.

Treatment of the PCa cell lines with the RAR antagonists led to necroptosis of CSC-like cells. Necroptosis is mediated by active PARP-1, and the PARP-1 inhibitor 1,5-dihydroisoquinoline blocked the actions of RAR antagonists. A significant aspect of the oncogenic action of active RARγ is that it blocks necroptosis. Necroptosis is viewed as a fail-safe cell death pathway for apoptosis-resistant cells [67] and one that defends against cancer [68]. The ability of the antagonists to drive necroptosis emphasizes an important avenue to treating cancer.

## 8. Perspectives

The findings from in vitro studies of PCa and breast cancer cells and in vivo studies of lung cancer support the further development of the RARγ antagonist AGN205728 and/or the pan-RAR antagonist AGN194310 for use to treat these cancers. The activity of AGN205728 and AGN194310 may extend to other solid tumor CSCs because RARγ is an oncogene for CRC, CCA, and HCC. However, there is need to test whether the antagonists are active against these cancer cells. To move the antagonists forward as drug candidates, there is the need to undertake preclinical studies using the 3-dimensional models that are available for PCa, human organoids, and xenograft models of, for example, breast and CRC cancer. These preclinical studies will reveal whether a projected therapeutic dose (from in vitro studies) eliminates CSCs, and is safe to use. Safety tests, including Ames mutagenicity, Chinese hamster ovary chromosomal aberration, and mouse micronucleus, are important to showing that there is no genotoxicity. 

The antagonists are highly selective for RARs. However, we do not as yet know the precise mode of action of RAR© in CSCs, nor how RARγ antagonism triggers necroptosis. There are two possible ways. Analysis of F9 embryonal stem cells, by integrative genomics, has revealed that RARγ regulates a large network of genes within stem cells [69]. RARγ is also essential for ATRA induced chromatin remodeling and the activation of transcription in embryonic stem cells [20]. Disruption to these processes may be the cause of RARγ antagonist driven necroptosis. RARγ antagonism may influence the cellular location of RARγ and this is important because cytosolic RARγ plays a role in controlling Riptosome (RIPK1/RIPK2)-mediated DNA damage-induced necroptosis when the cellular inhibitor of apoptosis is blocked [70,71]. Therefore, a more complete understanding of the precise mode of action of RAR© within CSCs and how RAR antagonism triggers necroptosis is needed. 

## Figures and Tables

**Table 1:** Binding affinities (ED_50_ in nM) of selected retinoids against RARs. Nuclear extracts were prepared from baculovirus infected Sf21 insect cells engineered to express either human RARα, -β or -γ. The equilibrium binding affinities of each retinoid analog were estimated by the [^3^H]-ATRA displacement method. ND, not conducted.

Retinoids	RARα	RARβ	RARγ	Classification
**RAR Agonists—Equilibrium Binding Affinities in nM**				
ATRA	ND	ND	ND	RARαβγ
AGN195183	20.1	>5000	>5000	RARα
AGN190168	>1000	14.2	135	RARβγ
AGN205327	3700	734	32	RARγ
**RAR Antagonists—Equilibrium Binding Affinities in nM**				
AGN194310	4.3	5	2	RARαβγ
AGN196996	3.9	4036	>10,000	RARα
AGN194431	300	6	20	RARβγ
AGN205728	2400	4248	3	RARγ

**Table 2:** Pan-RAR and RARγ antagonists are potent inhibitors of the growth of flask cultures of PCa cells * mean of the IC_50_ values obtained for the AGN194310 pan-RAR antagonist when tested against primary cells from 14 patients.

Cells	AGN194310 pan-RAR Antagonist IC_50_ Values	AGN193109 pan-RAR Antagonist IC_50_ Values	AGN193776 pan-RAR Antagonist IC_50_ Values	LG100815 pan-RAR Antagonist IC_50_ Values	AGN205728 RARγ Antagonist IC_50_ Values
***PCa cells***					
DU-145	5. 0 × 10^−7^ M			1.8 × 10^−6^ M	6.0 × 10^−7^ M
LNCaP	4.0 × 10^−7^ M	4.2 × 10^−7^ M	3.9 × 10^−7^ M	5.2 × 10^−7^ M	4.5 × 10^−7^ M
PC-3	3.5 × 10^−7^ M	6.8 × 10^−7^ M	5.7 × 10^−7^ M	1.0 × 10^−6^ M	4.7 × 10^−7^ M
Patients’ cells	4.7 ± 2.1 × 10^−7^ M *				3.0 × 10^−7^ M
***Non-malignant prostate cells***					
Prostate epithelial	1.0 × 10^−6^ M	1.4 × 10^−6^ M	1.1 × 10^−6^ M	>1 × 10^−5^ M	7.2 × 10^−7^ M
RWPE-1					2.3 × 10^−6^ M

**Table 3:** Pan-RAR and RARγ antagonists are potent inhibitors of colony formation by PCa cell lines.

PCa Lines	AGN194310 pan-RAR Antagonist IC_50_ Values	AGN205728 RARγ Antagonist IC_50_ Values	AGN196996 RARα Antagonist IC_50_ Values	ATRA pan-RAR Agonist IC_50_ Values
DU-145	34 × 10^−9^ M	60 × 10^−9^ M	>1 × 10^−5^ M	4.0 × 10^−7^ M
LNCaP	16 × 10^−9^ M	55 × 10^−9^ M	>1 × 10^−5^ M	3.2 × 10^−7^ M
PC-3	18 × 10^−9^ M	50 × 10^−9^ M	>1 × 10^−5^ M	4.2 × 10^−7^ M

## Data Availability

Not applicable.
